# Transforming the Adaptation Physiology of Farm Animals through Sensors

**DOI:** 10.3390/ani10091512

**Published:** 2020-08-26

**Authors:** Suresh Neethirajan

**Affiliations:** Ajna Consulting, 42 Edwards Street, Guelph, ON N1E 0B3, Canada; sneethir@gmail.com; Tel.: +1-226-979-3147

**Keywords:** adaptation physiology, sensors, precision livestock farming, wearable animal sensors, animal biometrics, animal cognition

## Abstract

**Simple Summary:**

Strategy for the protection and welfare of farm animals, and the sustainable animal production is dependent on the thorough understanding of the adaptation physiology. Real-time, continuous, and precise measurement of the multi-dimensions and complex intricacies of adaptive capacity of farm animals namely the mental, behavioral, and physiological states are possible only through the sensor-based approaches. This paper critically reviews the latest sensor technologies as assessment tools for the adaptation physiology of farm animals and explores their advantages over traditional measurement methods. Digital innovation, diagnostics, genetic testing, biosensors, and wearable animal devices are important tools that enable the development of decision support farming platforms and provides the path for predicting diseases in livestock. Sensor fusion data from a multitude of biochemical, emotional, and physiological functions of the farm animals not only helps to identify the most productive animal but also allows farmers to predict which individual animal may have greater resilience to common diseases. Insights into the cost of adoption of sensor technologies on farms including computing capacity, human resources in training, and the sensor hardware are being discussed.

**Abstract:**

Despite recent scientific advancements, there is a gap in the use of technology to measure signals, behaviors, and processes of adaptation physiology of farm animals. Sensors present exciting opportunities for sustained, real-time, non-intrusive measurement of farm animal behavioral, mental, and physiological parameters with the integration of nanotechnology and instrumentation. This paper critically reviews the sensing technology and sensor data-based models used to explore biological systems such as animal behavior, energy metabolism, epidemiology, immunity, health, and animal reproduction. The use of sensor technology to assess physiological parameters can provide tremendous benefits and tools to overcome and minimize production losses while making positive contributions to animal welfare. Of course, sensor technology is not free from challenges; these devices are at times highly sensitive and prone to damage from dirt, dust, sunlight, color, fur, feathers, and environmental forces. Rural farmers unfamiliar with the technologies must be convinced and taught to use sensor-based technologies in farming and livestock management. While there is no doubt that demand will grow for non-invasive sensor-based technologies that require minimum contact with animals and can provide remote access to data, their true success lies in the acceptance of these technologies by the livestock industry.

## 1. Introduction

Adaptation physiology or acclimatization is defined as an individual organism’s biological response to environmental stress. Adaptation can be broadly classified into genetic (generational or long-term) and non-genetic (phenotypic or short-term) responses to a stressor [1]. Under chronic stress experienced over several generations, the animal’s acclimatization response becomes genetically “fixed,” making the animal adapted to its environment.

The physiological and behavioral processes adopted by farm animals in response to environmental changes are not only crucial for their survival, but frequently also affect the profitability and productivity of livestock systems. “Farm animals” is a term used to describe a group of animals housed together in a barn or an animal husbandry. These animals are typically raised for the commercial utility of produce such as dairy, leather, meat, eggs, wool, etc. Livestock must face the multipronged challenge of physical, chemical, nutritional, and thermal stress [2]. Stressors or the challenges are several and may or may not have a direct influence on the animal performance. Factors that act as stressors and thereby influence livestock productivity include age, breed, geographical location, water availability, nutrient availability, photoperiod, environmental conditions, interactions with humans, and management practices [3]. Stressors trigger physiological mechanisms that allow animals to maintain physical equilibrium and homeostasis [4]. Farm animals respond to environmental stressors by altering physiological parameters like rectal temperature and respiration rate, drooling, panting, sweating, heart rate variability, and decreased feed intake [5].

In order to determine which technological advances can improve both livestock productivity and animal welfare, it is crucial to measure the physiological parameters or adaptation physiology of farm animals. This emphasis on measurement is driving the development of specific sensor-based technologies that can monitor these physiological changes. Quantitation of stressors in farm animals often involves invasive techniques that require animal restraint or close contact between animals and humans, which can increase the animals’ stress levels [6]. These techniques are also time-consuming, subjective, and labor-intensive, making them unsuitable for a rational evaluation of stress in farm animals [7].

Smart systems such as sensor technology play an important economic role in this area [8], offering considerable long-term financial advantages. The economic welfare modelling framework is a powerful tool for reinforcing policy decision-making in appraising alternate scenarios for animal diseases. Sensing platforms and tools are helpful in disease prevention and control strategies, giving them an important role in advancing public health policy [9].

The economic impact of livestock disease is wide-reaching and multifaceted—direct costs of a disease include production losses in addition to the costs of treatment and preventative care. Furthermore, sick animals have a lower economic value. Common treatment practices have a critical financial impact on the farmers and can impact long-term herd structure as a consequence of the outbreak. In terms of production parameters, as estimated in a previous study [10], milk production in cattle with Lumpy skin disease is reduced by up to 65% during the acute phase and 35% upon the cattle recover from the disease, revealing the disease’s protracted and marked negative impacts. These economic losses can be largely avoided with the implementation and integration of remote sensor technology or automated technologies in agricultural and livestock practices.

Livestock welfare in farms remains a major concern; in order to protect livestock from poor welfare situations, it is imperative to interpret their behavior as well as their cognitive needs and capacities [11]. The recent COVID-19 pandemic particularly demands minimal contact with farm animals, and the current situation is only expected to increase the demand for the use of sensor-based technologies and processes that can protect the health of both farm personnel and livestock. Driven by an interest in animal welfare, research has gathered speed in the development of new sensing approaches and biosensing methods for non-invasive assessment of physiological stress response in animals [12]. For instance, remote sensing-based technologies and image processing are being widely researched to aid in the detection of animal health problems and stress levels [13]. With respect to assessing adaptation physiology in farm animals, sensor technology is considered to be pivotal as it can assist in acquiring time series of behavioral and physiological data. These sensors include biosensors and wearable technologies, which are based on advanced statistical and computer science methods that can be used to predict and assess adaptive responses and resilience in farm animals.

Automated remote monitoring and detection of animal welfare indicating parameters using real-time analysis of sounds, images, videos, and data tracking for body and body weight conditions may improve biological metrics in livestock [14]. Remote sensor devices such as microphones, cameras, accelerometers, and thermometers can provide credible information when their data is merged with individual animal identification and referenced observations and incorporated in algorithms [15].

Sensors in animal health management can help facilitate timely diagnosis and follow-up treatment for sick farm animals [16]. The use of sensor-based technologies enables early identification of disease symptoms and subsequent disease management, minimizing the economic losses associated with infection spread in the herd. These are vital for the survival of any business [17]. Moreover, these sensors can be linked to cell phones and other mobile devices so that data can be analyzed and recorded automatically and remotely. The aim of this paper is to critically review sensor technologies as assessment tools for the adaptation physiology of farm animals and explore their advantages over traditional measurement methods. Furthermore, the review aims to highlight the challenges involved in the deployment of sensing technologies, especially regarding their applicability in farm settings.

### Literature Collection Methodology

The literature cited in this paper were collected using the Google scholar and the Web of Science tools. To showcase the leading-edge research in this field and to ensure the recency and narrow down the search, the author restricted the search to papers published only in the past 5 years. Keywords used were, animal sensors; livestock wearables; animal physiology and sensors; farm animal sensing technology; facial recognition and animals; precision livestock farming and sensors; adaptation physiology measurement. Both the Boolean and individual searches were conducted as part of this study. Dairy cattle, pigs/swine, chicken and poultry birds, and sheep were primarily focused as part of the consideration of the common livestock. The number of papers cited in this review is 143, with 36 that were published before 2015. The papers published before 2015 were included as the information on the sensing technologies for farm animals were scant, and to emphasize the content on the need for sensors in the adaptation physiology of animals.

## 2. Assessing Adaptation Physiology Parameters

**Figure 1:**
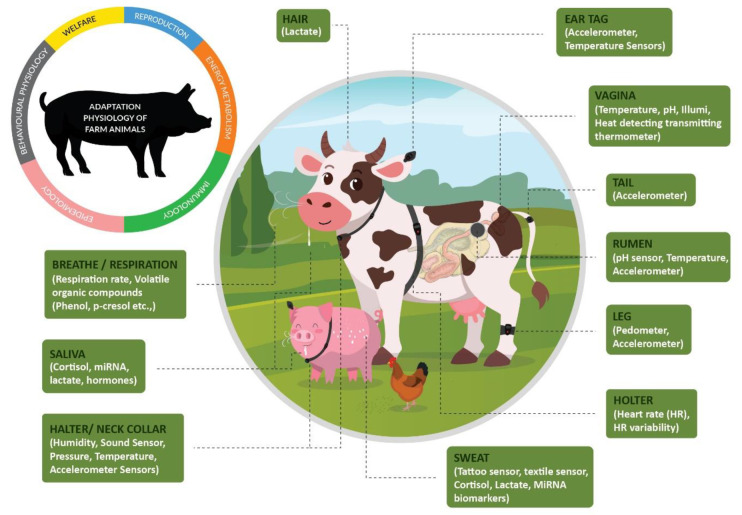
Wearable sensors and novel biomarkers for transforming the adaptation physiology of farm animals.

Precision Livestock Farming (PLF) is a tool for active livestock management with a focus on improving animal welfare and health and enhancing the economic, social, and environmental sustainability of livestock farming. The importance of well-managed animal welfare is not limited to ethical viewpoints; it is also crucial to realize a more efficient process of producing animal products. The metabolic energy balance in a homeothermic living organism includes several different components: basal metabolism, the thermal component, the physical component related to movement or delivering power, the production term (meat, milk, eggs), and the mental component. When applying stress-monitoring techniques originally developed for humans, animal frustration can be monitored in real time. This indicates that real-time animal welfare monitoring based on physiological signals is, in fact, a realistic pathway (Figure 1). Parameters such as temperature, heart rate, peripheral vasodilation, respiratory rate, sweat production, basal and energetic metabolisms, feed intake, diseased conditions, and sound all respond to stressful conditions [16]. Farmers can more quickly detect livestock health problems by evaluating their animals’ physiological responses via measurements of the temperature in body parts, respiration rate, heart rate variability, body weight, feed, emotions, non-invasive biomarkers and water intake, activity, and movement.

### 2.1. Body Temperature

An animal’s ability to maintain homeothermy indicates that the animal is better adapted to the climatic variations of its environment. Tolerance of higher temperatures, for instance, is identified by animals’ capability to dissipate excess body heat and keep their standard body temperature within the limits of homeothermy [3]. For this reason, monitoring changes in animals’ body temperature can help determine their physiological stress responses to their environment. As an example, it has been shown that broilers in high and medium-density housing have higher body, wing, head, neck, and skin temperatures than those housed in lower densities [18]. These results can be used to estimate potential stressors for farm animals. The cloacal temperature of a bird is typically measured by inserting digital thermometer into the bird’s cloaca [19]. This invasive procedure requires that the animal be manually restrained and handled [20], which would lead to stress-induced hyperthermia. Shortcomings of these conventional temperature measurement methods make them not practical for regular heat stress monitoring [21]. For an accurate, reliable, and continuous measurement of body temperature, farmers can deploy biologgers such as telemetry devices or the radio-telemetry data loggers by integrating with halters or collar/leg sensors for larger animals such as cows, pigs, and horses. Body temperature measurement with thermal infrared sensors is considered safer and more efficient than conventional techniques for livestock including broilers and poultry birds [22]. Infrared thermometry is non-obstructive in nature, which makes it suitable for assessing animal stress. Furthermore, the use of digital thermography (thermographic camera) makes it possible to detect even minimal temperature variations [23].

#### Advantages of Sensors in Assessing Body Temperature

Body temperature can be efficiently and accurately determined with non-invasive infrared technique [24]. In broilers, for instance, the temperature of the region around the eye is considered to be indicative of the total body temperature because of lack of feather insulation around the eye region [25]. All objects produce radiant heat in the infrared region of the electromagnetic spectrum. As bodies have temperatures above absolute zero, they emit radiation that forms an electromagnetic spectrum that can be absorbed by other surrounding bodies [26]. Thermal infrared (TIR) sensors generate these images (thermograms) by capturing the infrared radiation emitted by objects. The information generated by these images is then determined by algorithms to detect the temperature change. Thermographic images may point to alterations in blood flow resulting from increased body temperature due to stressful environmental conditions. These changes can be related to blood flow and heat transfer in animals [26]. The temperature of the eye area measured by TIR sensors has been shown to correlate significantly to changes in core body temperature [6]. Temperature measurements from different body parts provide information about animal health and allow for timely decision-making for livestock welfare (e.g., isolating animals with higher body temperature or managing the internal temperatures of animal housing units).

### 2.2. Respiration Rate

Respiratory rate (RR) is one of the variables used to assess the physiological adaptability of animals [27]. In response to alterations in environment, such as transport of cattle by road, animals’ respiratory rates increase to maintain homeothermy. Evaporation is a key regulatory response for animal body temperature [27]. RR has been shown to be affected by high milk yield, pregnancy, forced activity, excitement, and pathological conditions [28]. An increased RR is a significant stress indicator, particularly in thermally stressed animals [29]. Thermal stress results in a reduction in milk production, animal fertility, and general welfare [30], so early detection of alterations in RR can help farmers take specific measures to prevent animals from stress, thereby avoiding production losses while addressing animal welfare.

Visually counting flank movements is the most common technique for assessing respiratory rates in cattle [31]. However, this is a labor-intensive and time consuming method, and the continuous assessment of RR in long-term studies can be difficult and physically demanding, eventually resulting in miscounts [28]. Misinterpretation of results can also occur from nonspecific flank movements that do not arise from respiration [32]. Moreover, the continuous presence of a person can cause the animals stress and influence their respiratory rates, misleading the measurements. Therefore, RR sensors were introduced to assess RR without causing stress to animals. The initial respiratory sensors tested on animals were designed for human application. These devices consisted of a belt attached around the animal’s chest (similar to a holter) that measured thoracic and abdominal movements. Another system to monitor respiratory rate is a laser distance sensor during milking [33]. Respiratory sensors have been reported to accurately monitor respiratory rates in cattle [28].

### 2.3. Sweating Rate

Sweating is a physiological mechanism to regulate body surface temperature. Sweat is generated by the sweat glands in the rainy and dry seasons. The maximum mean values are reported in the mornings and afternoons during dry season, which aids heat dissipation by evaporation; maximum sweating capacity is achieved under high temperatures and low humidity, when the sweat glands are stimulated by increased blood volume to the epidermis [27]. The composition of sweat could be helpful in determining the internal physiological changes or disturbances in an animal. Sweat glands on pigs are considered non-functional as they are not stimulated by heat stress. Imperceptible perspiration in the form of water vapor may have biomarkers such as miRNA (micro Ribonucleic acid) or micro-concentrations of cortisol or hormones. Validated biomarkers from the sweat of dairy cows, pigs, and other farm animals are not yet available and would open up new avenues of non-invasive sensing.

#### Immunosensors

Immunosensors can target biological fluids like saliva and sweat instead of blood, making these sensors less stressful and invasive for animals. Immunosensors provide highly sensitive and specific assessment of hormones like cortisol and lactate in animal biological fluids using label-free electrochemical and chronoamperometric methods. For instance, a mobile, handheld potentiostat integrated with Bluetooth communication and power source can be used for point-of-care applications. Bioengineers have designed sensors with a sandwich-like structure that can contain the sensing mechanisms, secure the biosensor to skin, and use capillary action to draw sweat or other fluids towards the sensing mechanism [34]. Overall, the immunosensor showed remarkable specificity and sensitivity in addition to its non-invasive and point-of-care capabilities, making the diagnostic tool a versatile sweat-sensing platform. Skin-deployable microfluidic platform provides a significant capability for on-the-go real-time collection and monitoring of sweat biomarkers. Resettable epifluidic sweat patches can be placed on the skin of pigs or the sweat-secreting glands of dairy cows to collect and analyze sweat composition as a visual indicating sensor [35].

### 2.4. Heart Rate Variability

The cardiovascular system is regulated by the autonomic nervous system and controls the physiological systems impacted by stress [36]. The vagal element of the autonomic nervous system regulates heart rate during stress in farm animals [37]. Heart rate increases when animals are subjected to heat above their tolerance limits [27], so changes in heart rate can indicate physiological and psychological stress, disease, and coping strategies in animals [36]. Conventionally, electrocardiogram (ECG) has been used to detect heart rate variability. Despite providing good signal quality, its long-term use for continuous heart rate monitoring is inconvenient as the wet electrodes must be in close contact with skin. Additional limitations include restriction of movement, allergic reactions, skin irritation, and signal degradation due to continuous contact with wet electrodes [38]. Although dry electrodes and non-contact electrodes are available, they require the use of a chest band to keep the electrodes in place. This can be inconvenient, especially for use in animals.

#### Role of Sensors in Assessing the Variability of Heart Rate

Photoplethysmography (PPG) is a non-invasive and cost-effective method for the detection of blood volume alteration. PPG is an optical measurement that uses infrared lights to detect changes in blood volume in the microvascular bed of tissue [39]. This contrasts with the electrocardiogram (ECG), which measures heart rate by placing electrodes on the chest of the target animal. Doppler radar-based sensing technologies include: ultra-wide band radar [40] and near-field coherent sensing. Machine-knitted washable sensor platform based on textile has been developed and validated for precise epidermal arterial pulse waves and respiratory signals simultaneously [41]. However, this tool has been demonstrated only for biomedical human applications and would need further modifications for adoption in livestock sector. Researchers have experimented with non-invasive wearable sensing systems composed of photoplethysmogram and electrocardiogram (ECG) for continuous monitoring of vital signs in companion animals [42], but these systems have yet to be fully explored in the livestock sector. Body size and the shape of animals, chewing habits of pigs, licking habits of cows, body weight, dust and the harsh barn and farm environments, and discomfort to animals are some of the factors that make the adoption of HR and RR wearable sensing systems challenging for animal farming (Table 1). Commercially available textile-based wearable sensors, such as Hexoskin, Biometric Shirt, Dinbeat UNO’s multiparameter harness-sensing tools developed for humans and pet animals for measuring heart rate variability, respiratory rate, and maximal oxygen consumption would find new avenues of application in livestock farming.

### 2.5. Feed and Water Intake Behavior

Studies on cattle behavior have indicated that illness causes animals to spend less time feeding and more time resting [43]. Feeding and ruminating behavior in cattle provides significant information about their productivity, health, and welfare [44]. Changes in ruminating and feeding timing of cows are indicative of an underlying alteration in their welfare and comfort [45]. It has also been reported that cows diagnosed with metritis spent less time ruminating than their healthy counterparts during the pre- and post-calving periods [46]. As alterations in feeding behavior could also be indicative of illness in animals, several sensing methods have been employed to measure feeding behavior.

#### 2.5.1. Sensors for Assessing Food and Water Intake in Animals

##### Wearable Accelerometers

Wearable accelerometers have been evaluated as a tool for measuring cow behavior [44]. HOBO accelerometers, which are attached to the jaws of cows, can record the time each animal spends grazing and ruminating [47]. Neck-mounted accelerometers, based on either a multi-class support vector machine (SVM) or a decision-tree algorithm [48], have been tested for the evaluation of bovine ingestive behaviors. Neck-mounted triaxial accelerometers have been validated to assess drinking events, along with the algorithm that can distinguish feeding from drinking from a water trough [17].

#### 2.5.2. Nose Band Sensors

A noseband sensor called RumiWatch was developed to monitor eating and ruminating activities in dairy cows [54]. Animals deal with stressors like social changes, environmental changes, satiety, and infection by changing their behavior. Monitoring this behavior on a large scale becomes impractical because of the manpower required for continuous monitoring of a large groups of animals. Wearable sensor technologies make it possible to simultaneously measure real-time physiological parameters in a herd on a large scale when coordinated with appropriate interpretations of outputs [44]. Thus, wearable sensor technologies have an edge over traditional herd-based approaches as the data from wearable sensors can be analyzed immediately, enabling quick reaction times.

#### 2.5.3. Water Sensors

Water flow sensors can monitor group drinking behavior in pigs and are considered more precise than experienced observers [65]. The issues and limitations of water sensors includes variable water flow rates, installing sensors in existing plumbing, short drinking bouts, unspecified drinking behavior when a snout is in an outlet, and wastage of water [66].

#### 2.5.4. Radio-Frequency Identification (RFID) Tags

The requirements of an RFID system include an RFID transponder or ear tag and an RFID antenna located at the drinking sink or feeding unit. RFID systems (at drinking and feeding areas) have been used to monitor the events and duration of feeding and drinking behavior of individual pigs [56]. High-frequency (HF), low-frequency (LF), and ultra-high-frequency (UHF) are the frequency ranges typically used for RFID tags. These systems can have significant differences in read ranges as well as varying responses to the influence of materials, especially water and metal [67].

#### 2.5.5. Combination of RFID and Accelerometers

A combination of stationary RFID, accelerometer, and water flow meter sensors has been used to measure beef cattle drinking behavior and herd water consumption in grazing systems. This approach was determined to be reliable in recording behavioral measures, such as the frequency, duration, and number of visits to the water point per animal as well as the duration of drinking events per animal visit [17].

#### 2.5.6. Multi Sensor Systems for Continuous Monitoring of Growth

Garrido-Izard and colleagues have monitored a combination of ear skin temperature sensors, body weight measurement, and the amount of feed consumed and the duration per animal in order to identify animal behavior changes based on the integration of their intake patterns and the thermal data. Animals with higher-temperature measurements showed less thermal variability, and vice versa. The study also showed that animals with less alteration in feed intake during breeding have higher efficiencies [68].

### 2.6. Activity, Movement, and Postural Behaviors for Analysis of Animal Welfare

Posture and lying behaviors can be automatically quantified to increase the productivity and welfare of domesticated animals because changes in posture and activity indicate health and welfare issues [69]. For instance, changes in behaviors like walking, standing, and lying can indicate sickness in cows [70]. Monitoring postural changes is important in assessing calf or swine wellness or painful conditions. Numerous studies have studied postural behavior differences in cows who receive painful stimuli [71].

#### 2.6.1. Sensors for Assessing Activity, Movement, and Postural Behaviors

##### Accelerometers

Accelerometers, electromechanical devices that measure acceleration forces, have proven very accurate in monitoring activity and movement in the animals. The use of accelerometers has recently been widely used to quantify or evaluate animal behavior [8], grazing behavior in cattle [72], lying behavior in sows and lameness in cows [73]. The accelerometer’s smaller size and the versatility of the data generated make these technologies effective for examining animal behavior in farm settings [74]. Accelerometers are considered to be most efficient in assessing activity in pigs. Increased activity is indicative of stress in pigs, while reduced activity has been associated with disease [75] or changes in the environmental conditions of a barn [76].

Analysis of data from two-dimensional accelerometers has revealed that the percentage of time spent standing increases in calves after castration [77]. In another study, it was shown that five days after castration, calves preferred more time in lying down and less time in walking activity [78]. The differences between these two studies could possibly stem from the duration of monitoring and a considerable time-dependent change in behavior.

The current animal welfare determining factors are mostly conducted at a single point in time (i.e., providing water and food and treating diseases or illnesses as they arise), these assessments are often considered insufficient. Pattison and colleagues have designed a methodology for the continuous monitoring of interaction between animals fitted with proximity sensors attached to neck collars. The resulting data and levels of serum cortisol concentrations are expected to provide a visual map of the social structures of each group. This will allow scientists to explore the potential of proximity sensors as a welfare monitoring platform for measuring an animal’s freedom to express its normal behavior in natural state [79].

Accelerometer sensors have been used to accurately measure active and not-active behaviors of tiestall-housed dairy cows [80] and predict the behavior of dairy cows from the signal pre-processing sensor data [81]. Lying behaviors in calves have also been analyzed after administering analgesic drugs. Lying behavior decreased in calves following the induction of experimental lameness using an amphotericin B synovitis-arthritis induction model [82]. Accelerometer sensors have also been effectively used in the regular assessment of behavioral changes in response to pain [71]. The use of two accelerometers can automatically quantify the lying behavior in free-farrowing sows; challenges of automation in the lying behavior have been addressed in both free-farrowing sows as well as sows housed in movement-restricting barn environments [73]. More recently, data from triaxial accelerometers has been used to define general behavior recognition framework in the form of a hybrid model combined with biomechanical principles and machine learning tools [83].

#### 2.6.2. Video Imaging for Behavior Analysis

In domestic animals, aggression between individuals is a serious stressor and cause of injuries. Research is increasingly exploring facial expressions as a non-invasive means of obtaining quantitative data on an animal’s emotional state. Camerlink and colleagues have demonstrated the use of video imaging in analyzing the emotional state of pigs in an event of aggression or confrontation in dyadic contest. Facial metrics can be a powerful way of measuring the aggressive intention of animals. Facial metrics after retreat and/or during the episode of aggression in pigs differed significantly from the facial features prior to and during aggression [84].

Mounting behavior, observed in both female and male pigs throughout their lifetime, is mostly prominent during estrus. This behavior is manifested as follows: a pig typically places two front hoofs on the head or the body of another pig in the lying position. Mounting behavior can cause epidermal wounds, lameness, and fractures, resulting in economic losses and decreased productivity. Li and colleagues have developed a learning algorithm that detects swine mounting behavior based on the visible light images to enable timely detection and intervention [85]. This method has demonstrated high accuracy, sensitivity, and specificity. However, the sample size of the research included only four mini pigs, so the system still needs to be validated on a larger scale.

#### 2.6.3. Pressure Mats

Pressure mats are composed of a series of array of pressure sensing components with the measuring frequency that enables the mats to differentiate the simultaneous impact of different limbs [55]. Systems from various pressure sensor manufacturers have been able to successfully assess locomotion in healthy cows, horses, and sheep [59,86,87]. These systems were capable of assessing lameness in cows [88].

#### 2.6.4. Pedometers

Pedometers objectively measure an animal’s total number of steps and the total distance travelled via an algorithm that calculates the steps from the raw data [71]. While pedometers are comparatively easy to deploy and use, there is considerable variation in the number of steps traveled by each calf on different days and environmental conditions. There might be an association between the distance traveled by calves and stressful and painful procedures; one study determined that calves traveled fewer steps for four days following castration [89], while another study showed the association between stress experienced by calves and the number of steps traveled following castration.

Stress influences the distance traveled by calves, though gender differences must be accounted for in such studies; it is observed that steers travel fewer steps per day than bulls [89]. Pedometers have reportedly been useful in intelligently designed experiments to investigate changes in behavior after the animals go through a painful experience. Pedometers have been demonstrated in identification of early lameness in dairy cattle, though a 15% decrease in activity was required in order to accurately detect 92% of the lame cattle [90]. Pedometers can be valuable tools for detecting and assessing musculoskeletal pain as they rely on the direct quantification of locomotion.

#### 2.6.5. Gait Measurement Using TIR (Thermal Infrared) Sensors

TIR and RGB (red, green, and blue) image based sensor data has reportedly been useful in evaluating the walking posture and gait analysis of cattle through recorded videos [91]. Changes in posture while walking can indicate skeletal problems in livestock, so TIR sensors can be implemented to judge animal well-being.

#### 2.6.6. Global Position Systems (GPS) and Real-Time Location Systems (RTLS)

Various research investigations in the last decade have demonstrated the advantage of GPS telemetry devices for assessing livestock behavior when used in combination with other sensors/devices [50]. This has been used to distinguish between activities or assess energy expenditure [53]. GPS collars with activity sensors form an efficient technique for simultaneously monitoring the movements of grazing livestock and inferring animal behavior [52].

RTLS have been developed to locate the position of an object anywhere inside a specific area. The design of an RTLS consists of a receiver positioned closer to the desired monitoring space, active or passive tags deployed on the target objects, and a hardware and software to receive and interpret positional data. Tags used with RTLSs are typically smaller in size and have a longer battery life than existing GPS systems [71]. RTLS technology has been investigated to assess the association between behaviors like distance travelled and duration of time spent at feed bunk with clinical illness scores. An association was also found between the distance travelled by calves and the level of lung consolidation by RTLS monitoring, which indicated that assessing movement can help evaluate the wellness status of livestock animals [92]. The associations derived from these studies strongly suggest that RTLS platform is a legitimate tool for producing quantitative measurements of cattle activity. They can be further helpful in evaluating significant changes in pain status or wellness in response to an intervention [71]. RTLS has the distinctive advantage in monitoring an animal’s location anywhere within the farm; therefore, it does not restrict assessment to only drinking and feeding behaviors.

### 2.7. Acoustic Variability

Vocalization process in animals happens through the active generation of sounds with organs to express distinct physiological status. Vocalization may be spontaneous or triggered by an external event [93], but their reflection of an animal’s inner state makes them an efficient tool for monitoring animal wellbeing, stress response, and interaction among species. Heat stress is an important environmental stressor affecting poultry production and welfare, specifically growth, egg production, and impaired poultry health. Changes in behavioral and physiological responses due to heat stress might manifest as distinct vocalizations such as gakel, squawk, and alarm calls [94]. Du et al. [95] have demonstrated a correlation between vocalization in egg-laying hens and the temperature humidity index (THI). The authors specifically concentrated on the quantitative measurement of frustration-related vocalizations (squawk, gakel, and alarm calls) and concluded that specific vocalizations such as poultry squawk and alarm calls are significantly correlated with THI. Furthermore, because of thermal inertia in a henhouse, which is a potential early warning detection tool to avoid lags in monitoring the ambient environment [95].

Acoustic monitoring (or vocalization measurement) is a non-invasive and an accurate method of measuring biological responses in livestock; it can be used as an indicator of animal well-being in precision livestock farming, which focusses on addressing animal welfare concerns by facilitating the automated, continuous monitoring of livestock and enabling timely and appropriate interventions [96].

#### Sensors for Vocalization

Changes in sound pressure can be transformed into electrical signals, which are then received by audio equipment and processed as digital signals using signal processing techniques through standard computers. The definite evaluation of audio data is typically based on spectral analysis, where acoustic signals automatically separate into bands of appropriate frequencies. This is then followed by subsequent processing [57].

**Figure 2:**
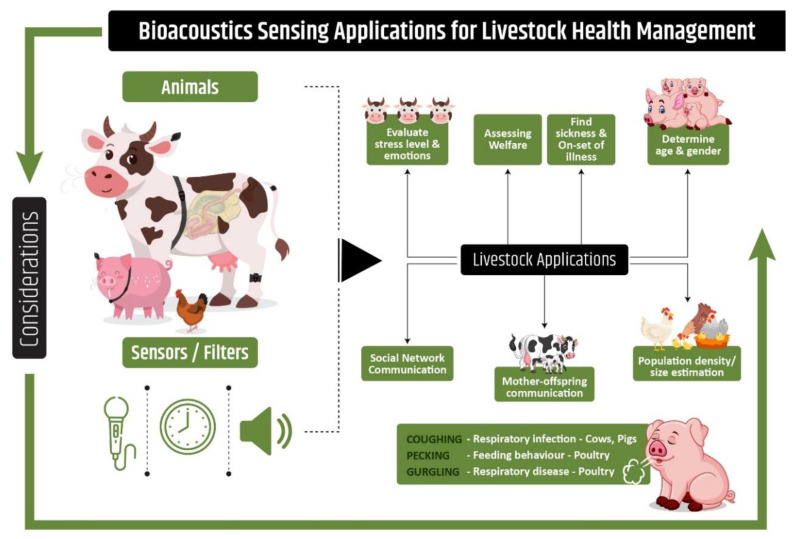
Bioacoustics applications in Precision Livestock Farming. Physiological parameters measurement through on-line sound analysis as a basis for health and welfare monitoring in pigs, cows, and poultry.

Vocalization in pigs can be used for several different purposes, such as identification of sex, age (Figure 2), and stress levels through a decision-tree to classify distress condition in pigs with an accuracy of 81.92% using the machine-learning technique [97]. In turkey farming, cannibalism is identified as a major problem resulting in animal stress and loss of productivity. Certain behavioral changes have been identified in animals prior to cannibalistic behavior, including pecking activity. Acoustic data analysis in combination with machine learning techniques has been tested as a tool to continuously monitor pecking activity for potential use on turkey farms [98].

### 2.8. Radar-Based Sensors for Activity Behavior

Currently, the activity-based sensors used in the animal farming industry are optical or animal-attached sensors. While sensors attached directly to animals are difficult to manage, the sensitivity of optical sensors decreases in the presence of dirt or changes in the ambient lighting conditions. In cases where spatial distribution of the animal movement or the activity is irrelevant, Manteuffel [99] has demonstrated that simple stationary sensors to measure animal activity can be beneficial due to easy signal interpretation requiring little computational power. Soon, doppler-based radar sensors will find new avenues in the dairy cow guided gate system to measure the cow’s heart rate and breathing pattern before entering the rotor milking machines.

### 2.9. Assessment of Reproductive Performance

Currently, there are no tangible ways to measure reproductive performance, especially with respect to uterine contractility and suitable diagnostic tests for the onset of labor in sows. Unsuitable uterine contractility can result in embryonic loss, miscarriages, ectopic pregnancies, and abnormalities of puerperium in sow [100]. One potential diagnostic tool is the measurement of bioelectric signals from swine uterus using electromyography and magnetomyography. To overcome the drawbacks in the crude uterine contraction information obtained through tocodynamometer or with an intrauterine pressure catheter, a 3D uterine electrical activation pattern measurement system has been demonstrated using electro-myometrial imaging (EMMI) [101].

EMMI surface electrical recording has been shown to be a safe, accurate, non-invasive, and feasible method to measure uterine contractions in sheep. Electrophysiological studies using electromyogram sensing systems by Domino et al. [102] demonstrated that myoelectric activity in various regions of sows’ reproductive tract can be used as a reliable measurement to assess pregnancy health as the contractility regulation was superiorly observed in the uterine horn tip. A tocodynamometer provides an indirect measure of the intrauterine pressure and is the current standard method to determine labor. A combination of electromyography and magnetomyography sensing platforms is expected to replace tocodynamometers in the near future. In a preliminary study, Brassel et al. [103] have evaluated the performance of automated health monitoring integrated with an accelerometer-based estrus detection system (ODS) for dairy cows on pasture. Although the ODS was able to flag health problems faster than human farmers, the use of ODS generated an extremely high rate of false positives [103].

### 2.10. Health and Disease

Absence of disease is an essential component of overall animal health and well-being. Disease, lameness, and limb disorders pose a significant challenge to the dairy industry today. Along with dairy cows, sheep flocks around the farm also face the most persistent and common health challenge of lameness. Lameness results in decreased milk production and is an important motivator for early culling of animals. Lameness is painful, and animals suffering from pain frequently diverge from their normal behavior by changing activity, gait, appetite, posture, and appearance [104].

#### 2.10.1. Sensors for Health and Disease

##### Bolus and Rumen Sensors

The amplitude and frequency of ruminal contractions in cattle are impacted by metabolic diseases (such as ruminal acidosis and hypocalcemia) and other diseases that cause fever or pain. Wireless intraruminal bolus sensors inserted through the esophagus have been developed to monitor the temperature and pH values of the rumen and reticulum [105]. The assessment process has been simplified by the advent of commercially available boluses for the measurement of reticuloruminal pH, where the boluses regularly transmit pH information wirelessly to a central processing region.

Wireless and indwelling sensors for rumen facilitates high-resolution pH measurements in determining the kinetic behavior and identification of rumen acidosis. The data from this analysis can assess the physiological status of the rumen and, therefore, the whole animal. While indwelling rumen pH sensors allow for continuous measurement of pH in individual animals, their application is limited; the differences in pH between the ruminal locations cannot be measured, and there is also substantial drift in the baseline sensor data from the non-retrievable sensors [106].

### 2.11. Metabolic Adaptations

The biochemical interactions within and outside the cellular environment lead to the end products, which are the metabolites. Comprehensive measurement of metabolites, called metabolomics, helps researchers gather accurate and sensitive data in order to better depict the phenotype [107]. Metabolomics is a non-invasive tool to identify phenotypes, phenotypic changes, and assessment of dietary responses [108]. Traditional methods of quantification of phenotypes, such as feed intake and residual feed intake, are time-intensive, costly, and require specialized equipment [109]. In contrast, phenotype/trait quantification using the metabolomics approach is cost-effective [110]. There remains a dearth of metabolomic studies on livestock, especially studies that utilize sensor technologies.

### 2.12. Use of Biosensors in Metabolomics

Biosensing technologies have started gaining attention in farmed livestock animal studies as they show promising potential to address the relevant issues of reliable tests, cost of equipment/devices, and early detection of disease/stress [14]. New and emerging biosensing technologies have the potential to improve livestock animal management and the associated factors [14]. These technologies monitor animal welfare by evaluating metabolic and stress biomarkers. The non-invasive early detection of stress by assessing physiological responses will help farmers take timely safety measures for animal health and welfare. Further advancements in biosensor technology shall generate new approaches for real-time evaluation of metabolic and physiological responses.

#### 2.12.1. Methane

A non-invasive technique to detect volatile organic compounds or a reliable validated biomarker allows farmers to responsibly assess animal stress and respond quickly. Such compounds can be identified in animal breath, skin, urine, feces, blood, and vaginal fluid [111]. Gaseous metabolites present in the breathing air of cattle include hydrogen, carbon dioxide, and volatile organic compounds such as phenol and methane. Methane emission in ruminants is an integral part of their energy metabolism and is thus a valuable indicator of their physiological state [112]. A study conducted to monitor methane emission in cows using a Fourier transform infrared (FITR) sensor assessed the breath of the animals and accurately measured the methane-carbon dioxide ratio [113]. Others have assessed methane levels in milk using the mid-infrared (MIR) spectra biosensor in cows [114]. The results have concluded that the extent of methane present in milk is directly dependent on the animal’s lactation stage. Phenol and p-cresol emitted from nasal secretions clearly indicated the bovine respiratory disease in cattle [115]. Real-time detection of volatile organic compounds using biosensors by integrating with the halter or the neck collar of cattle or pigs would serve as a non-invasive, portable disease-sensing tool.

#### 2.12.2. Glucose

The concentration of metabolites in tears is correlated to their concentration in the blood. Thus, tears can be used for the non-invasive continuous evaluation of metabolites. Currently, there are no devices or sensors available for measurement in livestock tear fluids as there are no scientifically validated biomarkers. This opens up new avenues of wearable ‘eye-based’ sensors for livestock.

#### 2.12.3. Hormones

Hormone levels related to the reproductive physiology of animals are usually determined to predict the animal’s reproductive state. The Herd Navigation system is a commercially available biosensor that can quantify levels of milk progesterone [116]. A handheld, smartphone-based, rapid on-farm progesterone immunosensor has been developed to monitor milk progesterone levels in cows [117], with a biosensor composed of a monoclonal anti-progesterone antibody (mAb) immobilized on an electrode. Others have also reported the use of immunosensors for the assessment of hormone levels in saliva, where the method is quick and non-invasive in nature.

#### 2.12.4. Pathogen/Virus

Farm livestock is often challenged by outbreaks of viruses like Bovine Herpes Virus-1 (BHV-1), foot-and-mouth disease virus (FMDV), and Bovine Viral Diarrhea (BVD) virus, to name a few. Various immunosensors have been built to determine the presence of these pathogens in animal sera [61], and these biosensors have produced faster results than conventional enzyme-linked immunosorbent assay (ELISA) and Polymerase chain reaction (PCR) based methods. Biosensor technology offers the advantage of being used in combination with other devices and being open to multiplexing, making it suitable for large-scale applications for agriculture and livestock.

### 2.13. Emotional Expressions

Specific internal or external stimuli trigger short-term, intense states known as emotions, which can lead to behavioral decisions and social interactions (i.e., approaching or avoiding the stimuli) [118]. Emotions have been shown to be of paramount importance in animal welfare, especially in the production chain of farm animals [119]. Studies have suggested that communication by vocal contagion [118], monitoring ear posture [120], facial expressions [121], and body language in livestock animals can provide a better understanding of their emotional adaptation [122]. However, this requires skillful, experienced workers and may provide erroneous diagnosis [123]. Researchers are evaluating coding systems that could provide objective readings of animal facial expressions instead of guessing the meaning of their expressions. A coding system precisely describes the meaning of different facial expressions such as squinting eyes, posture of ears, eye white region, or pursing lips when an animal feels a particular emotion. Such techniques have been developed and tested on domestic animals in some studies. For instance, EquiFACS has been developed as an anatomy-based objective tool to determine the systematic recording of facial expressions and pain scoring in horses [60]. However, the scientific community has still not come up with a detailed validation of the outcomes of such a system in farm animals. It is hypothesized that in the future, developers could create a smart phone app that could decipher the emotional status of an animal when the user held the phone in front of the animal’s face.

## 3. Limitations of Sensing Technologies

Although sensor technology has been proven to provide accurate and reliable results in farm animal assessment, it poses a unique set of challenges. For instance, optical sensors such as video cameras, infrared thermography, or depth cameras are commonly used to automatically assess general activity. These stationary optical devices are prone to reduction in sensitivity due to dirt and dust [124].

Depth cameras are susceptible to disturbances from heat sources including sunlight (in the case of infrared-based systems) or the color and structure of the animals’ feathers or fur [125,126]. Radiation measurements rely on the analysis of the animal’s emissivity and conductivity values.

Furthermore, infrared sensors need specialized IR translucent window cover materials in comparison to plastics or steel based covers, as the sensors are prone to damage by physical forces due to the harsh farming environments [99]. Studies that have attempted to show the correlation between broiler outer body temperature and core body temperature used IR cameras under well-controlled research conditions [127]. The high-quality IR cameras used in such studies are expensive and remain impractical for commercial applications [127].

PPG has contact requirements, so its advanced version, photoplethysmographic imaging (PPGI), is being considered for use in livestock. Unlike the contact-based requirements in PPG, PPGI is camera-based, low-cost, non-contact, and convenient. It is also preferable over a PPG sensor in areas of hygiene, animal welfare, and practical deployment for housed animals [38]. However, even PPGI technique is not completely free of loopholes. It requires that subjects face the camera and remain motionless during recording. PPGI signals are also prone to illumination variations and motion-induced artifacts (Table 2), especially while dealing with webcams through ambient light [128].

### Use of Big Data in Smart Farming Solutions

The era of “big data” is interdependent on the development of novel sensing technologies that allow for rapid and inexpensive collection of observations and data [128]. The trend towards agricultural applications of big data techniques and methods is not strictly about primary production; farmers also aim to improve the efficiency of the entire supply chain and alleviate concerns related to food security [129].

The extent of literature on the use of big data in smart farming is limited in peer-reviewed literature, but the commercial viability of Internet of Things (IoT) and new technologies for wireless connectivity is generating huge amounts of data that can be used for end-to-end livestock management [129]. Furthermore, as all this data is available in real time, it can be used to support decision-making capabilities that were incomprehensible before.

The scope of the use of big data in adaptive physiology includes automatic phenotype identification of animal breeds using computer vision and machine learning techniques [130], genomic prediction, and disease detection for sub-clinical mastitis applying machine learning-based prediction platforms on milking datasets to study the best predictive models of sub-clinical mastitis [131]. Other methodologies exploring the detection of sub-clinical mastitis include the use of cytometric fingerprinting and machine learning [132]. Big data and machine learning have been used for the analysis of animal behavior [133]. Computer vision-based methods have been employed for automatic recognition in commercial farms using spatial and temporal information of the nursing behavior of animals and individual pig recognition, with accuracy rates of 96.7% [134,135].

Machine learning tools have been used to evaluate cattle behavior and feed intake using predictive clustering trees (PCT) [136] and by combining accelerometer data with machine learning to analyze cattle behavior including walking, grazing, ruminating while standing, resting while standing, ruminating while lying, and resting while lying [81]. While this method resulted in excellent behavior prediction accuracy, the machine struggled to differentiate cow postures based on data from a single accelerometer. Other methodologies to determine feed intake include the adoption of a machine vision system for feed intake by individual animals based on deep convolutional neural networks (CNNs) models, and a low-cost RGB-D (Red, Green, Blue, Depth) camera [137]. Big data and machine learning are being evaluated in several other areas as standalone methods or in combination with conventional sensors to evaluate adaptive behavior in livestock.

These technologies can determine several other physiological parameters, such as heat stress in dairy cows. This method is considered superior to current approaches of determining stressors as several machine learning algorithms consider nonlinearity in the data, thereby removing the subjectivity [138]. Big data has been used in the analysis of animal behavior, quantitative risk assessment for animal disease transmission, and implementation of practices for risk-reduction [139]. In near future, big data application would be commonplace in analyzing animal behavior and welfare. Big data’s true potential in practical terms will undoubtedly require close collaboration in fields such as computer science, engineering, mathematics, statistics, and the livestock industry to enable the development of cutting-edge approaches to analyzing large quantities of heterogeneous data [140]. Predictive machine learning approaches, in combination with sensor-based data, can prove invaluable in addressing the challenges ahead in animal sciences.

## 4. Economic Viability of Adoption of Sensors for Adaptation Physiology of Animals

Some of the costs associated with implementation of sensors technology for investigation and measurement of adaptation physiology of farm animals are expenses involving software, hardware sensing electronic components, data storage and information costs, data processing, and learning costs for the producers to develop the management framework. Size of the farm greatly influences the adoption and profitability of using sensors technologies as the cost/benefit estimations need a specific minimal farm size to depreciate the investments. The benefits of sensing technologies that enable real time data collection on the animal behaviors and the adaptation physiology are beginning to be validated. Benefit–cost ratio, internal rate of return, payback period, net present value, and milk production measures in evaluating the economic value of the investment in precision livestock farming platforms such as the Automated Estrus Detection tools [141] assured farm profitability and enhancement in production efficiency. Cost–benefit analysis of four fictive pig and dairy farms by Hammer et al. [142] showed that the ultra-high frequency and radio frequency identification tags (UHF-RFID) sensor platform in the simultaneous detection and hotspot monitoring of fattening pigs and dairy cows is economically advantageous. However, this study was conducted based on only fictive farms, and did not include additional benefits such as animal welfare indicating parameters for supporting quality marketing, and the advantages associated to traceability programs.

## 5. Future Perspectives

The advent of sensor technologies has revolutionized the assessment of livestock behavior and stress responses. Sensor-based technologies have contributed immensely to minimizing the stress on animals, improving animal welfare, and consequently preventing economic losses. Early detection of physiological responses can help farmers take targeted measures to alleviate the strain on their animals, improve animal welfare, and prevent performance losses by predicting potential disease outbreaks. Sensor technologies have an edge over traditional assessment methods as they are timesaving and can automatically take measurements at desired time (Table 1). These efficient technologies aid in the evaluation of certain responses that can then be used to precisely estimate physiological states such as stress, welfare, fertility, health, metabolism, and disease.

On the technological front, a number of novel sensor-based methodologies are still in the explorative phase of development. Some promising sensor candidates include microRNA-based sensors for detecting bovine respiratory syncytial virus, sensors for the detection of salivary hormones such as Luteinizing Hormone for the detection of bovine estrus, and electrochemical sensors to detect antibodies against influenza A and B, to name a few. These rely on highly specific biomarkers for specific physiological conditions. None of the currently available commercial devices and sensing systems provide a combination of size, functionality, and wearability that meets the requirements of the livestock sector or allows for the movement of animals and the simultaneous measurement of physiological parameters such as respiratory and heart rate. This gap calls for research in the design and development of sensing technologies for next generation of precision livestock farming.

It should be noted that some of the precision livestock farming sensing platforms do not require internet connectivity and can possibly function by using Bluetooth and/or radio frequency spectrum. These sensors may be used in isolation in which encrypted data can be collected from multiple animals and barn systems, compiled, and sent to a local computational platform for data processing [143]. With the advent of Digital Twins technologies in livestock farming, real-time data transmission collected by the multitude of sensors from farms is one of the basic design requirements. Moreover, the OIE—World organization for animal health, strongly advises real-time detection and transmission of animal health and epidemiological data from the sensors and devices [14] to producers, inspection, and government agencies. Hence, internet connection in livestock farms becomes necessary. The limited internet connectivity and the data moving capacity for both rural and urban farms remain a bottleneck for adoption of sensor-based platforms in investigation of adaptation physiology of farm animals.

Globalization, the post-Covid-19 world, rising per-capita incomes, human population growth, ecological pressures, and global warming are some of the many important parameters that will influence the role of technology in the field of adaptation physiology for farm animals. It is evident that Covid-19 is building individual and societal resilience as it forces entire industries to find new and innovative solutions for farming, livestock, and other industrial sectors. The emphasis on reducing animal experiments for research purposes and limiting physical contact with animals is expected to fuel future research and development as well as create new commercial applications for sensor-based technologies in the agricultural and livestock sectors.

## 6. Conclusions

Sensor-based technology can assess many biochemical, metabolic, physical, and immunological parameters related to adaptation physiology in farm animals. These include measurements of heart rate variability, respiration rate, body temperature, sweating rate, metabolism, health and diseases, vocalization, activity, movement, postural, feed and water intake behavior, and emotional contagion. The presented critical review illustrated the promising outcomes of sensor-based technology in farm animals as an innovative approach for maximizing animal welfare and providing better alternatives for gauging animal health and response. In addition, sensor-based technologies help in the recognition of robust breeds (i.e., animals with better adaptation capabilities and stress response).

Despite promising outcomes, the use of novel, highly precise sensor-based technologies face several challenges that must be addressed in future research. These key challenges include the validation of large-scale machine learning techniques and issues related to the sensitivity of conventional sensor-based methodologies. Another important challenge will be imparting the necessary skillset to farmers in rural areas, so they are equipped and willing to maximize their use of the available technology. Educating the end-users of sensor-based technology in the use of information technology-based sensor devices is the only way these innovative new developments can reach their full potential.

## Figures and Tables

**Table 1:** Outcomes of using sensor technology for assessing adaptation physiology of farm animals.

Farm Animal	Physiological Parameter/Biological Sample Involved	Sensing Technology	Outcome	Reference
Cattle	Rutting span behavior and back posture	Video and Photo sensors	Health and reproductive assessment	[6]
Cattle	Eye orbital area temperature	Infrared thermography	Non-invasive and automated detection of bovine respiratory disease onset	[49]
Cattle	Grazing behavior	Motion sensors	Accurate classification of grazing and rumination	[50]
Cattle	Feeding behavior differences associated with lameness	Neck-mounted mobile sensor	Detection of differences in feeding behavior that are associated with lameness	[51]
Cattle	Grazing, standing, and ruminating	Collar, halter, and ear tag sensors	Classification accuracy achieved by different sensor placement	[52]
Cattle	Feeding, ruminating, and other activity	RumiWatch halter and Accelerometer	Collar mounted accelerometer accurate than RumiWatch noseband sensor	[44]
Cattle	Respiratory rate	Differential pressure sensor	Suitability of the device as a respiratory rate sensor	[28]
Cattle	Drinking and water intake behavior	RFID, water flow meter, and accelerometer sensors	Reliability of a combined approach recording several behavioral measures	[17]
Cattle and sheep	Eye and muzzle temperature	Digital infrared thermal imaging	Alternatives to using vaginal or rectal temperature	[26]
Cows, goat, and sheep	Body acceleration	Accelerometers	Potential for estimating energy expenditure in grazing farm animals	[53]
Cows	Grazing and ruminating times	Tri-axial accelerometers	An alternative to visual observations	[47]
Cows	Ruminating and eating behavior	Noseband pressure sensor	An alternative to direct observation	[54]
Pigs	Gait and locomotion	Pressure mat	Objective way to analyze and quantify gait	[55]
Pigs	Drinking behavior	RFID system	Accuracy in assessing drinking behavior of individual pigs	[56]
Pigs	Conformation and posture	Accelerometer	Characterization of posture changes	[57]
Broiler	Body (rectal) temperatures as well as head, neck, wing, body, and shank surface temperatures	Infrared camera	Association between body surface body temperature and stocking density rate in broilers	[18]
Broiler	Broiler head and flock temperature	Infrared thermal camera	Achieving a higher identification accuracy	[58]
Horses	Walking and trotting	Pressure sensor	Simultaneous quantification of differences in hoof contact area and limb loading	[59]
Horses	Facial expressions	EquiFACS	Nostril dilator showed a 92% correct classification in duration in pain/no pain	[60]
Cattle	Blood samples	Biosensor	Bovine Herpes Virus	[61]
Cows	Blood and serum	Microfluidics based biosensor	Ketosis, Beta hydroxybutyrate	[62]
Chicken	Hen movement and activity	Three-axis accelerometers	Impact of Northern Fowl Mite on the welfare	[63]
Chickens, Cattle and Pigs	Screaming in pigs, tail biting in pigs, distress calls and alarm calls in chicks, cough sounds in cattle	Microphones, audio recording sensors	Monitoring aggressive interactions, context-based cattle calls labelling, classification of distress vocalizations	[64]

**Table 2:** Challenging factors in deploying sensor technology for animal farming.

Challenges	Affected Systems
Dirt and dust	Optical sensors
Sunlight	Infrared-based sensors
Color and structure of animals’ fur and feathers	Time of flight sensors
Physical force	Infrared sensors
Expensive instrumentation	Photoplethysmographic sensor (PPGI)
Motion-induced artifacts	Webcam and PPGI
Networking in rural area farms	Wi-Fi and Bluetooth based devices
